# To Close, Observe, or Reconstruct: The Third Way of Managing Dialysis Fistula Aneurysms in Kidney Transplant Recipients

**DOI:** 10.3390/jcm10194567

**Published:** 2021-09-30

**Authors:** Krzysztof Bojakowski, Aneta Gziut, Rafał Góra, Bartosz Foroncewicz, Stanisław Kaźmierczak, Dominika Kasprzak, Jolanta Małyszko, Piotr Andziak

**Affiliations:** 1 2nd Department of Vascular Surgery and Angiology, Centre of Postgraduate Medical Education, 01-813 Warsaw, Poland; k_bojak@yahoo.com (K.B.); rraaff@interia.pl (R.G.); dkasprzak0507@gmail.com (D.K.); pmandziak@gmail.com (P.A.); 2 Department of Invasive Cardiology, Warsaw of Centre of Postgraduate Medical Education, 01-813 Warsaw, Poland; anetagziut@poczta.onet.pl; 3 Department of Immunology, Transplantology and Internal Disease, Medical University of Warsaw, 02-091 Warsaw, Poland; bartosz.foroncewicz@wum.edu.pl; 4 Faculty of Mathematics and Information Science, Warsaw University of Technology, 00-661 Warsaw, Poland; st.kazmierczak@gmail.com; 5 Department of Nephrology, Dialysis and Internal Disease, Medical University of Warsaw, 02-091 Warsaw, Poland

**Keywords:** kidney transplantation recipient, immunosuppression, dialysis fistula, complication

## Abstract

Background: The management of patent dialysis fistulas in patients after kidney transplantation (KTx) is controversial—the options that are usually considered are the fistula’s closure or observation. Many complications of dialysis fistulas occur in patients after KTx, and immunosuppression increases the risk of fistula aneurysms and hyperkinetic flow. This study aimed to evaluate the results of dialysis fistula aneurysm treatment in patients after KTx and to compare them to procedures performed in an end-stage renal disease (ESRD) dialyzed population. Methods: We enrolled 83 renal transplant recipients and 123 ESRD patients with dialysis fistula aneurysms qualified for surgical revision to this single-center, prospective study. The results of the surgical treatment of dialysis fistula aneurysms were analyzed, and the primary, assisted primary and secondary patency rate, percentage and type of complications were also assessed. Results: For the treatment of dialysis fistula aneurysms in transplant patients, we performed dialysis fistula excisions with fistula closure in 50 patients (60.2%), excision with primary fistula reconstruction (*n* = 10, 12.0%) or excision with PTFE bypasses (*n* = 23, 27.7%). Postoperative complications occurred in 11 patients (13.3%) during a follow-up (median follow-up, 36 months), mostly in distant periods (median time after correction procedure, 11.7 months). The most common complication was outflow stenosis, followed by hematoma, dialysis fistula thrombosis and the formation of a new aneurysm and postoperative bleeding, infection and lymphocele. The 12-month primary, primary assisted and secondary patency rates of fistulas corrected by aneurysm excision and primary reconstruction in the KTx group were all 100%; in the control ESRD group, the 12-month primary rate was 70%, and the primary assisted and secondary patency rates were 100%. The 12-month primary, primarily assisted and secondary patency rates after dialysis fistula aneurysm excision combined with PTFE bypass were better in the KTx group than in the control ESRD group (85% vs. 71.8%, 90% vs. 84.5% and 95% vs. 91.7%, respectively). Kaplan–Meier analysis showed a significant difference in primary patency (*p* = 0.018) and assisted primary (*p* = 0.018) rates and a strong tendency in secondary patency rates (*p* = 0.053) between the KTx and ESRD groups after dialysis fistula excisions combined with PTFE bypass. No statistically significant differences in patency rates between fistulas treated by primary reconstruction and reconstructed with PTFE bypass were observed in KTx patients. Conclusions: Reconstructions of dialysis fistula aneurysms give good long-term results, with a low risk of complications. The reconstruction of dialysis fistulas can be an effective treatment method. Thus, this is an attractive option in addition to fistula ligation or observation in patients after KTx. Reconstructions of dialysis fistula aneurysms enable the preservation of the dialysis fistula while reducing various complications.

## 1. Introduction

It is still controversial to close or preserve a functioning arteriovenous fistula after successful kidney transplantation (KTx) [1,2,3]. There are arguments for both options—a patent dialysis fistula, especially hyperkinetic, increases cardiac output and pulmonary pressure, increasing cardiovascular risk, and fistula closure leads to a clinically significant reduction in left ventricle mass [4] and could potentially prevent cardiological complications. On the other hand, the risk of kidney transplant failure and return to dialysis due to end-stage graft insufficiency, despite continuous progress, is still significant [5]. It is sometimes extremely difficult and not always feasible to obtain a well-functioning dialysis fistula in patients returning to hemodialysis due to kidney transplant failure [6,7]. Additionally, the effect of dialysis fistula closure on transplanted kidney function has not been definitively determined; in some studies, it led to the deterioration of renal function [8], but in others, it led to improved urine volume, renal creatinine clearance and proteinuria [9].

In patients after kidney transplantation, in addition to ligation or fistula preservation and observation, it is also possible to perform a dialysis fistula reconstruction. A significant percentage of dialysis fistulas in patients after kidney transplantation develop various complications, e.g., stenosis, aneurysms and thrombosis. Different fistula redo procedures can be performed to eliminate these complications, including optimizing fistula function, reducing hyperkinetic flow and widening stenoses that threaten thrombosis. However, immunosuppressive drugs may increase the risk of various complications after surgical procedures. This influence can depend on various factors, including the type of procedure, the extent of the operation, the presence of the arteriovenous anastomosis or the use of a vascular prosthesis. The results and complications of such treatment are not yet known.

Our study aimed to assess the effects of redo procedures of dialysis fistula aneurysms after kidney transplantation: closure, primary reconstruction and reconstruction with prosthesis.

## 2. Materials and Methods

### 2.1. Patients

Inclusion criteria: All kidney recipient patients (>12 months after transplantation, with continuous immunosuppression, not requiring dialysis at the time of procedure) and who were qualified from 2012 for dialysis fistula aneurysm surgical treatment till 2020 were included in the study (at least 3 months of follow-up). The indications for dialysis fistula aneurysm reconstructions in this study included the following: hyperkinetic blood flow (>2000 mL/min) with heart failure, completed or threatening thrombosis, skin necrosis, bleeding, a threatening rupture, potential lack of accessible cannulation sites in patients with a kidney graft increasing insufficiency requiring preparation for return to hemodialysis. From the beginning of the study, patients suspected of heart failure—due to blood flow>2000 mL/min or clinical symptoms—were examined with echocardiography. Later, we implemented obligatory echocardiography examination, and now in our center echocardiography is performed in all patients during qualification for dialysis fistula surgery. The following were considered echocardiographic heart failure: systolic failure—EF <45% ejection fraction, dP/dt <1000 mmHg/s (left ventricular systolic pressure change rate).

Exclusion criteria: lack of informed consent or appropriate cooperation of the patient. To assess the results of dialysis fistula redo procedures in kidney transplant recipients and especially the impact of immunosuppression, we used the same operations performed in end-stage renal disease patients (ESRD) on chronic dialysis for correction of dialysis fistula aneurysms. The control group consists of dialyzed patients from 2012 who were qualified for dialysis fistula aneurysm treatment. The indications for surgery were identical to those in the study group. Patients were selected for some analyses to eliminate the potential impact of differences in the frequency of known risk factors for complications (diabetes, thrombosis, BMI, surgery type). Out of 11 dialyzed patients (included in the study to the control group) who had their fistula removed, 10 were converted to CVC (9 were permanent due to cardiac failure, 1 had a new peripheral fistula after 3 months). Before removal of the dialysis aneurysm, 1 patient had previously developed a peripheral fistula on the opposite forearm. One of our top priorities for dialysis access is to avoid the use of CVC. In the case of reconstruction of extensive dialysis fistula aneurysms, we usually perform the procedure in two stages to avoid the use of CVC.

### 2.2. Ethics Statement

This study was conducted following the Helsinki Declaration and was approved by the Ethical Committee of the Central Clinical Hospital Ministry of Internal Affairs (49/2010). Written informed consent was obtained from all patients.

### 2.3. Fistula

Doppler ultrasound (DUS) examination of the dialysis fistula (11L linear probe, GE LogiQ GE HealthCare, USA) was performed before and after surgery. The dialysis fistula’s maximal diameter, aneurysm length, stenosis or thrombus presence and blood flow characteristics were studied. Based on the DUS examination, the possibility of fistula reconstruction, the preferable type of surgery, was also assessed.

Qualification for fistula closure or reconstruction depended on several factors, the most important of which were the transplanted kidney function stability and the expected duration of remaining without the need for dialysis, fistula hemodynamics (especially the presence of hyperkinetic flow), the coexistence of other fistula pathologies (e.g., stenosis or thrombus) increasing the risk of complications and patients’ expectations and needs.

### 2.4. Surgical Procedure

All types of these operations are routine procedures described previously [10]. All procedures were performed by two vascular surgeon consultants (KB, RG), each with experience in >1000 dialysis fistula operations.

In brief, in the dialysis fistula aneurysm excision, the fistula was dissected from the anastomosis site, the entire expanded fistula segment from the anastomosis was removed and attempts were made to maintain the proximal non-widened part just above the first patent side branch, considering that such a vein could potentially be used in the future for fistula creation, and runoff from the branch is sufficient for prevention of the left section of the vein thrombosis.

Dialysis fistula aneurysm primary reconstruction—in the case of indications for the preservation of the fistula and favorable anatomical conditions, a primary reconstruction was performed after aneurysm excision by a new arteriovenous anastomosis—in some cases, was combined with the proximalization of arteriovenous anastomosis or with the conversion in radio-cephalic fistulas from end-to-side to end-to-end anastomosis, which enables the use of a proximal radial artery segment for reconstruction.

For dialysis fistula excision with polytetrafluoroethylene (PTFE) in the case of indications for fistula preservation, the fistula aneurysm was excised and replaced by a prosthesis PTFE (W.L. Gore, Flagstaff, AZ, USA) placed in another subcutaneous channel. For reconstruction, we preferably used 6 mm standard wall PTFE, but in selected cases, due to different dialysis fistula anatomical configurations, we also used 8 mm PTFE and tapered 4–7 mm PTFE graft.

### 2.5. Follow-Up

After surgery, patients were routinely followed up according to the local protocol, monitored, and all complications and redo procedures were noted and analyzed. Control clinical and DUS examinations were performed 1 month and every 6 months after dialysis fistula reconstruction, and patients with significant complications were qualified for secondary procedures. The minimal follow-up in the observed group was 3 months, and the median follow-up was 36 (mean ± SD, 35.9 ± 20.85) months.

### 2.6. Statistics

The survival curves were calculated using the Kaplan–Meier method. The number of patients who remained at risk for an event at a particular time point is shown below the x-axis. All data were calculated using Python programming language and the Statsmodels, SciPy, lifelines, and Matplotlib packages. We conducted separate tests for the binary and continuous variables to determine the statistical differences between pairs of groups. For the first type, to check the equality of the proportions in the analyzed groups, we applied Fisher’s exact test (the Fisher–Irwin test). The Mann–Whitney test was applied for the continuous variables to determine whether the two groups followed the same distribution. We deemed two samples to have significantly unequal proportions (in terms of binary variables) or significantly different distributions (in terms of continuous variables) if the *p*-value obtained from the test was less than 0.05. Log-rank analysis was performed to determine significance in patency between groups. Groups of people with and without immunotherapy significantly differ in terms of patients’ characteristics. Thus, we specified the most important features (“THROMBOSIS”, “THROMBOSED FISTULA”, “URGENT”, “EXCISON+PRIMARY RECONSTRUCTION”, “EXCISION”, “EXCISION+PTFE”, “BMI”, “FALSE ANEURYSM”, “DIABETES”) for which we did not want to have any significant difference (*p*-value higher than 0.05). In the preliminary analysis, we found that thrombosis, urgent surgery, concomitant diabetes and type of surgery significantly influenced the results of dialysis fistula aneurysm operations. Hence, we attempted to select groups comparable in these parameters for further analysis. *p*-value higher than 0.05 for each feature is a necessary condition; nevertheless, we aimed to achieve the highest possible values. Analysis of all possible subgroups is infeasible (exponential time complexity). Thus, we applied a random selection process with 100,000 iterations. In each iteration, 30 patients with and 30 patients without immunotherapy were randomly drawn. Finally, two 30-person groups for which statistical tests (Mann–Whitney for the continuous and Fisher’s exact test for binary variables) returned the highest minimal *p*-value among selected features were chosen for further analysis. The minimal *p*-value equals 0.24, yielding no significant difference between the two groups.

## 3. Results

### 3.1. Dialysis Fistula Aneurysm Correction in Kidney Renal Transplant

From March 2012, we performed surgical corrections of dialysis fistula aneurysms on 83 kidney transplant patients. The patient cohort consisted of 53 males (63.9%) and 30 females (36.1%), with a median age of 57 (range: 29–77) years. Eighteen patients (21.7%) were diabetic, 74 (89.2) had hypertension, 24 (28.9%) had coronary disease, four (4.8%) had previous myocardial infarctions, two (2.4%) had cerebrovascular disease, one (1.2%) had another peripheral aneurysm and two (2.4%) had previous venous thrombosis episodes. Patient characteristics and used immunosuppressive drugs are shown in Table 1. None of the kidney recipients operated for dialysis fistula aneurysm were chronically treated with anticoagulants, but there were two such patients in the control group.

### 3.2. Dialysis Fistula

Corrected dialysis fistulas were located on the upper arm in 41 patients (49.4%). Thirty-six (43.4%) patients had forearm autogenous radio-cephalic direct wrist fistulas, 31 patients (37.3%) had autogenous brachio-cephalic upper arm direct fistulas, three patients (3.6%) had autogenous brachio-basilic upper arm transposition fistulas, seven patients (8.4%) had a Gracz (brachial artery-perforating vein) fistula and six patients (7.2%) had a composite prosthesis–upper arm vein fistula. The aneurysm diameter was 27.2 ± 9.22 mm, and length 144.6 ± 77.63 mm (mean ± SD). Dialysis fistula aneurysm heterogenicity and characteristics (type, location, diameter, length, Balazs-Bjork [11] and Valenti [12] scales) show the extent of the aneurysm and the coexistence of pathologies (stenosis, wall-adherent thrombus or thrombosis and the presence of pseudoaneurysms), as shown in Table 2. Balazs-Bjork [11] and Valenti [12] scales are described below:

Valenti scale for dialysis fistula aneurysm classification:

1a: Dilated along the length of the vein, 1b: post-anastomotic aneurysm; 

2a: Classic “camel hump”, 2b: combination of type 2a and 1b;

3: Complex;

4: Pseudoaneurysm [12].

Balazs-Bjork scale for dialysis fistula aneurysm classification:

1—Without stenosis and thrombosis;

2—With hemodynamic significant stenosis (≥50%) (2a) in inflow artery, (2b) at arterial anastomosis, (2c) along cannulation zone, (2d) in the central vein;

3—With partial thrombosis occluding ≥50% of the lumen;

4—With complete thrombosis [11].

The mean time between creating a dialysis fistula to its reconstruction was 84.6 (range, 15–264) months. Indications for fistula reconstruction, in several cases multiple, to correct a dysfunctional dialysis fistula included fistula thrombosis in four patients (4.8%), hyperkinetic flow with cardiac overload in 48 patients (57.6%), risk of rupture in nine patients (10.8), no need for a fistula and patient’s willingness to remove in 47 patients (56.6) and potential problems with cannulation sites in eight patients (9.6%).

In the study group, we performed dialysis fistula aneurysm excisions with fistula closure on 50 patients (60.2%, in one patient it was combined with the creation of the new dialysis access in different localization), excision with primary fistula reconstruction on ten patients (12.0%) and excision with PTFE bypass on 23 patients (27.7%).

Postoperative complications occurred in 11 patients (13.3%) during a median 36-month follow-up. The most common complication was outflow stenosis noticed in six patients (7.2%). Other complications included hematoma (*n* = 3, 3.6%), dialysis fistula thrombosis and the formation of a new aneurysm (*n* = 2 each, 2.4%), and postoperative bleeding, infection and lymphocele (*n* = 1 each, 1.2%).

A flowchart detailing the two groups of patients and the type of FAV complication is given below in Table 3.

Some patients developed more than one complication. Given in patient number (and percentage).

#### 3.2.1. Comparison of Dialysis Fistula Reconstruction Results in Patients after Kidney Transplantation in Comparison with Dialyzed Patients

In the first stage, the treatment of dialysis fistula aneurysms in a cohort of 83 patients after kidney transplantation was compared to the results of 123 patients on chronic dialysis undergoing dialysis fistula aneurysm correction at the same time. KTx patients were significantly younger than patients from the ESRD group (53.2 ± 12.97 vs. 65.9 ± 16.09 years, *p* < 0.05), respectively. Regarding the fistula type, KTx patients compared to the ESRD group more often had a fistula on the forearm (49.4% vs. 23.0%), radio-cephalic (43.4 vs. 18) and Gracz fistulas (8.4 vs. 1), and less often the brachio-basilic (3.6 vs. 17) and combined PTFE segment with arm vein (7.2 vs. 28) fistulas, respectively. In the KTx group, significantly less frequently, the indication for correction was fistula thrombosis; in these cases, the indication was severe pain, inflammation or a significant risk of recurrent thrombosis episodes. Repair in the KTx group was carried out for aneurysms of smaller diameter (27.2 ± 9.22 vs. 32.5 ± 10.51 mm, *p* < 0.05), but longer (144.6 ± 77.63 vs. 91.2 ± 64.99 mm, *p* < 0.05) than in patients on dialysis. Much less frequently, dialysis fistula aneurysms in KTx patients were complicated and associated with wall-adherent thrombus, narrowing (25.3 vs. 43.1%), thrombosis (3.6 vs. 20.3%) and rupture risk (10.8 vs. 24.4%), but were more often longer and embraced the entire fistula length (Valenti’s scale, 1) (63.8 vs. 20.1%). In dialysis patients, aneurysm excision with dialysis fistula removal was more common (60.2 vs. 8.9%), and aneurysm excision combined with PTFE bypass was performed less often (27.7 vs. 76.4%). The percentage of primary dialysis fistula reconstruction in both groups did not differ statistically (12.0 vs. 8.1). We also performed other procedures in the ESRD group, e.g., patch plasty in several pseudoaneurysm cases or endoluminal angioplasty.

The patients from the KTx group significantly differed from the ESRD group in morphology and biochemical parameters, as presented in Table 4. The results indicate that the complication rate was significantly lower in the KTx group than in the ESRD group (13.2 vs. 50.4, *p* < 0.05), and in particular in the KTx group, significantly less secondary thrombosis (2.4% vs. 33.3%, *p* < 0.05), new aneurysm formation (2.4% vs. 12.2%, *p* < 0.05) and outflow vein stenosis (8.4% vs. 33.3%, respectively) occurred relative to the ESRD group. Due to the significant difference in some features between the KTx and ESRD groups, we filtered and selected patients to reduce these differences in the next stage. We aimed to obtain comparable groups with differences (*p* < 0.02) for features particularly important for the results and occurrence of postoperative complications (the types of operations performed, diabetes, BMI, urgent indications and fistula thrombosis as an indication for surgery). We successfully selected 30-people groups, which enabled us to perform the analysis (it was not possible for groups of 40 patients). This significantly reduced the number of analyzed patients but still exceeded the number of patients in groups of individual operations. However, still, the selected groups differed in patients’ age: the group after KTx was statistically significantly younger (52.0 ± 13.74 vs. 71.6 ± 12.30 years old), had a higher percentage of fistula on the forearm (50% vs. 13%) and aneurysms were smaller in diameter (27.6 ± 9.38 vs. 33.8 ± 10.17 mm, *p* < 0.05) and longer (141.2 ± 89.27 vs. 99.0 ± 67.55 mm, *p* < 0.05) than in the ESRD group. In the selected groups, we found four complications (13%) in kidney recipient patients and nine complications (30%) in the dialyzed patients; this difference *p* = 0.209 was not statistically significant.

#### 3.2.2. Comparison of the Results of the Dialysis Fistula Aneurysm Excision without Reconstruction between the Kidney Transplant Patients and the ESRD Group

Dialysis fistula excision without fistula reconstruction was performed on 50 patients in the KTx group and 11 in the ESRD group. KTx patients were significantly younger than ESRD group individuals (53.1 ± 12.57 vs. 68.7 ± 17.23 years, *p* < 0.05). There were no statistically significant differences in the diameter of the aneurysm between the groups. In the KTx group, aneurysms were statistically significantly longer (162.5 ± 68.80 vs. 122.2 ± 84.66 mm, *p* < 0.05), less frequently coexisted with stenoses (10% vs. 27.3%) and were at risk of rupture (2% vs. 27.3%). There was no statistical significance of complication risk—in both groups, one complication occurred: lymphocele in the ESRD group and hematoma in the KTx group.

#### 3.2.3. Comparison of the Results of the Dialysis Fistula Aneurysm Excision Combined with Primary Reconstruction between the Kidney Transplant Patients and the ESRD Group

Dialysis fistula excision with primary reconstruction was performed in ten patients in both kidney transplant recipients and dialysis patient groups. KTx patients were significantly younger than the ESRD group individuals (51.2 ± 11.75 vs. 64.00 ± 13.39 years, *p* < 0.05). There were no statistically significant differences in the diameter (29.0 ± 10.42 vs. 35.1 ± 5.09 mm, *p* = 0.075), length (89.0 ± 66.77 vs. 71.6 ± 35.84 mm, *p* = 0.455) or coexisting pathologies rates between the groups. Median follow-up in the KTx and ESRD groups was 39 and 34.5 months, respectively. There was no statistical significance of postoperative complication risk: in the KTx group, there were complications in two patients (two outflow stenosis, in one patient coexisting with a new aneurysm), and in the control group, complications occurred in five patients (two inflow stenosis and three outflow stenosis). The 12-month primary, primarily assisted and secondary patency rates of fistulas corrected by aneurysm excision and primary reconstruction in the KTx group were 100% each, and in the ESRD group 70.0%, 100% and 100%, respectively. The 24-month primary, primary assisted and secondary patency rates of fistulas in the KTx group were 100% each, and in the ESRD group were 66.7%, 80% and 90%, respectively. The 36-month primary, primary assisted and secondary patency rates of fistulas in the KTx group were 83.3% each, and in the ESRD group were 40%, 57.1% and 57.1%, respectively. The types of surgical procedures and outcomes in both groups are given in Table 5.

**Figure 1:**
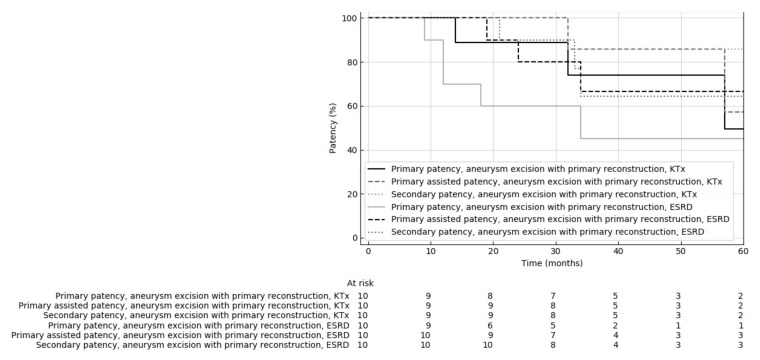
Comparison of primary, primary assisted and secondary patency rates of dialysis fistulas reconstructed by fistula aneurysm combined with primary reconstruction in kidney transplant patients (KTx) and dialyzed patients with end-stage renal insufficiency (ESRD).

Kaplan–Meier analysis showed no significant difference between primary, assisted primary and secondary patency between the KTx and ESRD groups (*p* = 0.307, 0.674, 0.386, respectively) (Figure 1).

Less than half of the complications in the study occurred within 1 year after reconstruction (the median time of complication onset was 12.67 months after surgery).

#### 3.2.4. Comparison of the Results of the Dialysis Fistula Aneurysm Excision Combined with PTFE Bypass between the Kidney Transplant Patients and the ESRD Groups

**Figure 2:**
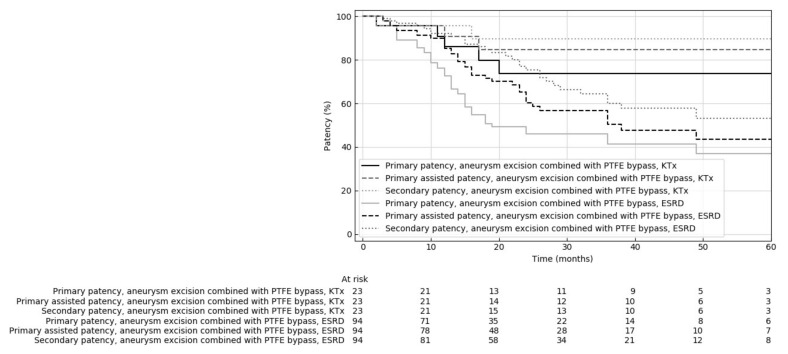
Comparison of primary, primary assisted and secondary patency rates of dialysis fistulas reconstructed by fistula aneurysm combined with PTFE bypass graft in kidney transplant patients (KTx) and dialyzed patients with end-stage renal insufficiency (ESRD).

Dialysis fistula excision with PTFE bypass reconstruction was performed on 23 patients in the KTx group and 94 in the ESRD group. KTx patients were significantly younger than ESRD group individuals (54.3 ± 14.40 vs. 66.4 ± 15.84 years). There were statistically significant differences in the location and type of treated dialysis fistulas. In the KTx group, the dialysis fistula was more frequently situated on the forearm than in the ESRD group (47.8 vs. 22.3%, respectively), and brachio-elbow fistula (0 vs. 19.1) was much rarer. The KTx group patients did not undergo surgery due to fistula thrombosis, which was often an indication of surgery in patients in the ESRD group (0% vs. 25.5%). In the KTx group, the diameter of the aneurysm was smaller (26.3 ± 9.47 vs. 32.3 ± 10.97, *p* = 0.04), and the length was greater (89.0 ± 66.77 vs. 71.6 ± 35.84 mm) than in the ESRD group, but these differences were not significant (*p* = 0.075 and *p* = 0.455, respectively). Regarding pharmacological treatment, antiplatelet drugs were used more frequently in the KTx group than in the ESRD group (21.7% vs. 5.3%). There was no statistically significant difference for postoperative complication risk: in the KTx group, there were complications in 34.8% of patients (four outflow stenosis, two hematomas, one lymphocele and one infection); in the ESRD group, complications occurred in 53.2% of patients (37 thrombosis, ten recurrent aneurysms, four hematomas, four inflow stenosis, 22 outflow stenosis, eight lymphocele, ten infections, of which one required prosthesis removal due to threatening septic bleeding, and one distal ischemia caused by stealing syndrome). Less than half of the complications in the study occurred within 1 year after reconstruction (the median time of complication onset was 12.67 months after surgery). The 12-month primary, primarily assisted and secondary patency rates in the KTx group were 85%, 90% and 95%, and in the ESRD group were 79.2%, 86.6% and 91.7%, respectively. The 24-month primary, primary assisted and secondary patency rates of fistulas in the KTx group were 68.8%, 80% and 86.7%, and in the ESRD group were 36.6%, 53% and 69.8%, respectively. The 36-month primary, primarily assisted and secondary patency rates of fistulas in the KTx group were 64.3%, 76.9% and 83.3%, and in the ESRD group, 26.6%, 37.9% and 50%, respectively. Kaplan–Meier analysis showed a significant difference in primary patency rates (*p* = 0.018), assisted primary (*p* = 0.018) and strong tendency in secondary patency (*p* = 0.053) between the KTx and ESRD groups (Figure 2).

#### 3.2.5. Comparison of the Results of the Dialysis Fistula Aneurysm Reconstruction Procedures—All Native Vessels vs. PTFE Bypass Creation in the Kidney Transplant Patient Group

**Figure 3:**
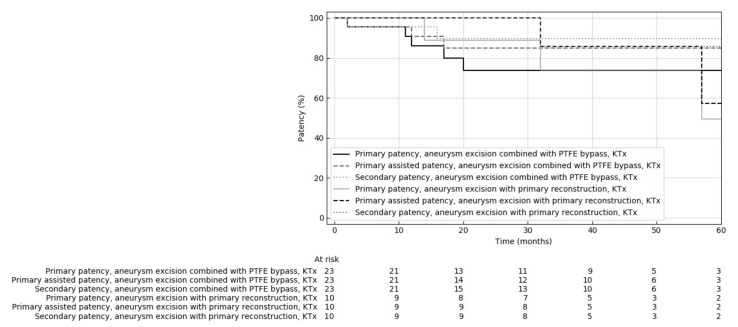
Comparison of primary, primary assisted and secondary patency rates of dialysis fistulas reconstructed by fistula aneurysm combined with primary reconstruction or PTFE graft in kidney transplant patients (KTx).

The use of a vascular prosthesis for reconstruction is potentially associated with an increased risk of various complications, including infection and stenosis in venous anastomosis. For this reason, in dialysis fistula aneurysm reconstructions, it is recommended to preferably use aneurysmorrhaphy or primary reconstructions without prosthesis rather than PTFE bypass. However, the latter reconstructions are commonly used for aneurysms associated with fistula stenosis and significant kinking. The impact of prosthesis use on the results of fistula reconstruction in patients after kidney transplantation is unknown. We compared the results of aneurysm excision combined with primary reconstruction (10 patients) with these reconstructions, joined with PTFE bypass creation (23 patients) in kidney transplant recipients. The characteristics of fistulas and patients did not differ significantly between the two groups; only aneurysm length was almost statistically significant in primary reconstruction and PTFE bypass groups (89.0 ± 66.77 vs. 135.0 ± 83.03 mm, respectively, *p* = 0.054). Differences were found in some features of morphology and biochemistry between the examined groups (Table 6). The risk of complications was not significantly higher in patients who underwent PTFE bypass 35% vs. 20% (*p* = 0.682) (complications were described in the previous sections). Kaplan–Meier analysis did not show statistically significant differences in primary, assisted primary and secondary patency after primary reconstruction compared to the PTFE bypass (*p* = 0.894, 0.841 and 0.970, respectively) (Figure 3).

Complications for the three interventions (fistula closure, fistula excision and reconstruction and excision with PTFE bypass) are as follows: complications occurred in two (20%) patients after primary reconstruction (two outflow stenosis, in one case coexisting with a new aneurysm), and in eight (34.75%) after excision combined with PTFE graft (four outflow stenosis, two hematomas, one lymphocele and one infection). Excision of dialysis fistula aneurysm in kidney recipients was the safest procedure—complications (hematoma) were found in only one patient (2%), as described in Section 3.2.2. Then, we compared the results of dialysis fistula excision combined with the primary reconstruction or PTFE bypass.

## 4. Discussion

It is known that the management of patent dialysis fistulas in patients after kidney transplantation (KTx) is controversial, and there are no uniform recommendations. In the United States, the proportion of patients who underwent fistula closure after KTx varied substantially between transplant centers, ranging from 0% (43.0% of centers) to >10% (11.0% of centers) [13]. In patients with well-functioning renal transplants, the dialysis fistula becomes useless and contributes to left ventricular hypertrophy, an increase in venous return and cardiac output. Recently, Rao et al. demonstrated that elective ligation of patent AVF in adults with stable kidney transplant function resulted in a clinically significant reduction in left ventricle (LV) myocardial mass [4]. Immunosuppression is a risk factor for fistula dialysis vessel dilatation and aneurysm formation, which often coexists with the hyperkinetic flow, which subsequently increases the risk of cardiological consequences of a patent fistula. On the other hand, dialysis fistula ligation has a negative impact because of an increase in diastolic pressure, total peripheral resistance and pulse pressure; moreover, it could favor LV concentric remodeling [2]. From a nephrologist’s point of view, there are also serious doubts that the ligation of a dialysis fistula may worsen the function of a transplanted kidney. Additionally, it is sometimes extremely difficult and not always feasible to obtain a well-functioning dialysis fistula in patients returning to hemodialysis due to kidney transplant failure [6,7]. This may be one of the reasons shown in current data that most patients with end-stage kidney transplant insufficiency are qualified for hemodialysis and frequently use dialysis catheters as vascular access against recommendation [14].

We believe that in addition to the ligation of the fistula or leaving it with the combination of its observation, it is also possible to perform a fistula reconstruction to eliminate various complications. In the past, various procedures performed on dialysis fistulas in patients after kidney transplantation have been described. Our previous work demonstrated the possibility of percutaneous angioplasty for the successful correction of dialysis fistulas stenosis in renal transplant patients [15]. Some studies have also shown the possibility of successfully reconstructing clotted dialysis fistulas in patients returning to dialysis due to increasing transplant renal insufficiency [6,7]. The current study indicates the possibility of reconstructing a dialysis fistula aneurysm in patients after kidney transplantation not requiring dialysis treatment.

Aneurysms are a common complication of dialysis fistulas, and they can complicate both AVG (estimated frequency 2–10%) and AVF (≈15%) [10,11,12]. The average incidence of dialysis fistula aneurysms is 0.04 cases per 1000 person-days (within a very wide range of 0–3.01). The frequency of the pathology described in the literature varies, depending on the definition of an aneurysm used and the method of fistula monitoring to detect this complication. Immunosuppression, as described in the text, is a significant risk factor for the development of dialysis fistula aneurysms. There are no unequivocal nephrological, transplant or surgical recommendations regarding the monitoring or management of detected dialysis fistula aneurysms. We estimate that the formation of a dialysis fistula aneurysm affects approximately 7–10% of dialysis patients in the dialysis centers cooperating with us. After the diagnosis of dialysis fistula aneurysm, some patients choose to follow up and postpone fistula reconstruction, sometimes agreeing to minimally invasive intravascular correction of the identified stenosis. Publicizing information in transplant centers of potential negative impacts of dialysis fistula aneurysms resulted in more frequent referrals of patients. The stability of kidney function causes patients and doctors of transplant centers to qualify the dialysis fistula aneurysm for earlier removal, and the poor function of the transplanted kidney leads to the assessment of the possibility of dialysis fistula reconstruction and improvement in its function.

Among the methods of dialysis fistula aneurysm surgical reconstructions that preserve patent dialysis access, primary reconstruction is much more beneficial, with a lower risk of complications and a better patency rate than PTFE bypass. However, this type of reconstruction is not always possible for anatomical reasons, including too-long aneurysms, concomitant stenosis and significant kinking. However, even dialysis fistula aneurysm excision combined with the formation of PTFE bypass is a procedure that can be successfully performed on patients after kidney transplantation. Previous studies have shown that corrections of dialysis fistula aneurysms using a vascular prosthesis have worse results than reconstructions using solely the patient’s own vessels (primary anastomosis or aneurysmorrhaphy). Reported primary patency 12 months after the excision of dialysis fistula aneurysm in combination with PTFE bypass is 40–71% [16,17].

Additionally, the use of a PTFE prosthesis due to frequent stenosis often requires subsequent balloon angioplasty. Such a scheme significantly increases the percentage of functioning patent fistulas in long-term observation. In our study, we did not observe a statistically significant difference in patency rates of fistulas reconstructed with or without PTFE prosthesis. Moreover, the patency of PTFE bypass performed in patients after kidney transplantation was significantly better than in patients on chronic dialysis. The use of immunosuppressive drugs (e.g., sirolimus) may reduce the risk of developing neointima and stenosis in fistula anastomoses, which is the main limitation of PTFE dialysis fistulas. The inhibition of neointima formation reduces the risk of various complications, especially thrombosis. Additionally, the risk of infection or septic bleeding associated with PTFE prosthesis infection in renal transplant patients receiving immunosuppressive drugs in our study was low and was not a significant problem.

Our study shows that the surgical correction of dialysis fistula complications, such as an aneurysm, combined with leaving patent dialysis access, is possible and effective in patients after kidney transplantation. This procedure should be considered as another option alongside fistula removal or monitoring.

The sole removal of a dialysis fistula in patients after a kidney transplant is not associated with a higher risk of complications than dialysis. In our group of patients, the risk of complications after fistula removal could have been increased by the extent of the surgery, which involved removing a long (median 17 cm length) fistula aneurysm. The risk of simple dialysis ligation of a non-widened fistula, which requires less preparation, should be lower. Limiting the procedure to fistula ligation with the preservation of the aneurysm in many cases leads to fistula thrombosis and inflammatory reaction and may even lead to pulmonary embolism. For this reason, dialysis fistula aneurysms qualified for closing should be removed, not ligated.

Our study shows that dialysis fistula aneurysms in kidney transplant recipients differ from those in dialyzed patients. A significantly lower percentage of stenoses, adherent thrombus and fistula thrombosis in the KTx group is caused by the selection and qualification of some patients to conventional treatment. However, smaller diameter and longer aneurysms, located more frequently on the forearm, indicate that the etiopathogenesis of aneurysm formation in renal transplant patients may be different than in dialysis patients. The most likely cause is the use of immunosuppression treatment. Previous studies have indicated that immunosuppression in organ transplant recipients increases the risk of dialysis fistula aneurysms, their growth and the risk of the rupture of abdominal aortic aneurysms [18,19,20]. The etiologic factors predisposing transplant patients to reduced vessel wall mechanical strength, aneurysm formation and expansion remain undetermined. In the case of dialysis fistula vessels, some immunosuppressants (e.g., tacrolimus) may affect the properties of smooth muscles, leading to wall weakness. In addition to the type of immunosuppressive medications, other factors, such as the frequency of rejection, infection, insulin resistance, lipid abnormalities, hypertension and other hemodynamic factors, may play a role [21].

Additionally, the types of operations performed on patients after kidney transplantation and dialysis patients were different in our study. In the group after KTx, fistula aneurysms were more often simply excised, and reconstructions aimed at preserving functioning fistulas were performed only on patients whose kidney function indicated the risk of having to restart dialysis or who wanted to have dialysis access secured.

In patients after kidney transplantation, no clear indications for the management of patent dialysis fistula exist; in most cases, leaving the dialysis fistula or its ligation are regarded as the only therapeutic options. Our work indicates the possibility of a dialysis fistula reconstruction, which allows the correction of its various pathologies while maintaining patency. We believe that the management of dialysis fistulas should be individualized to the specific patient and his or her clinical situation. The best approach to dialysis fistula management in renal transplant patients should be further investigated. The optimization of management should consider several factors related to transplanted kidney function, coexisting diseases, life expectancy, history of dialysis access and the function of the current dialysis fistula and potential dialysis access alternatives. Further, multi-center studies should be conducted to determine the management recommendations for dialysis fistulas in patients who have had a kidney transplant.

### Limitations

Our study was a single-center study, but the large number of patients included should be emphasized. Another important problem with our study that might affect the results is the significant differences between the KTx and ESRD groups. This applies especially to the patients’ age and the types of operations performed. By dividing the groups based on the procedure types, we obtained groups large enough to perform the analyses while eliminating differences in the percentage of types of operations. Furthermore, some differences between the groups were evened out by selection performed in additional analyses, which led to a remarkable reduction in the number of compared groups. As we have indicated earlier, we believe that multi-center and randomized studies rationalizing the management of a patent dialysis fistula in patients after kidney transplantation should be carried out. Not all patients were initially assessed routinely with echocardiography, and we are currently conducting a study on the effects of a hyperkinetic fistula on the heart, but the populations studied are not the same.

## 5. Final Considerations

### 5.1. What Is Already Known about This Subject

There are no clear recommendations regarding the management of a patent dialysis fistula after successful kidney transplantation.

A patent dialysis fistula after kidney transplantation unnecessarily increases cardiac output, which may lead to pulmonary hypertension; additionally, immunosuppressive treatment increases the risk of dialysis fistula dilatation, leading to hyperkinetic flow.

Dialysis fistula closure decreases the cardiologic problems of increased cardiac output, pulmonary hypertension. Still, it can potentially lead to the deterioration of the transplanted kidney’s function and problems with vascular access if kidney transplant failure occurs.

### 5.2. What This Study Adds

In addition to simple ligation of the dialysis fistula or its preservation and observation, it is possible to perform secondary reconstruction of the dialysis fistula in patients after kidney transplantation.

The results of dialysis fistula reconstruction in patients after kidney transplantation are equal or better than in patients on dialysis, depending on the type of surgery.

Differences in patency rates after primary dialysis fistula reconstruction with re-anastomosis and its removal and bypass creation with PTFE graft are not statistically significant.

### 5.3. What Impact May This Have on Practice or Policy

Our study shows that dialysis fistula reconstruction to maintain a functioning fistula is a safe and effective method in patients after kidney transplantation.

The reconstruction of the dialysis fistula aneurysm, even with a vascular prosthesis bypass in kidney transplant recipients, is associated with a lower risk of complications than dialysis patients.

Fistula reconstruction to maintain a functioning fistula should be considered another option, fistula ligation or maintenance and follow-up.

## 6. Conclusions

It is possible to perform a safe reconstruction of a dialysis fistula, correcting its complications and leaving a functioning dialysis fistula in kidney transplant recipients. This is an additional option for the management of dialysis fistulas in patients after KTx. This is particularly important in patients with poor function of a transplanted kidney with a high risk of returning to dialysis and patients with potential problems of creating new dialysis access. The treatment results and the risk of complications are not higher in patients after kidney transplantation compared to patients on dialysis.

## Figures and Tables

**Table 1:** The kidney transplant recipients with corrected dialysis fistula aneurysm—patient characteristics and used immunosuppressive drugs.

Variables	*n* = 83
sex (men/women)	53/30
age (mean, range) (years)	53.2 (29–77)
Body Mass Index (mean, range)	25.9 (15.76–35.46)
smoking (number, %)	8 (9.6)
hypertension (number, %)	74 (89.1)
diabetes mellitus (number, %)	18 (21.7)
coronary disease (number, %)	24 (28.9)
myocardial infarction (number, %)	4 (4.8)
peripheral artery disease (number, %)	4 (4.8)
cerebrovascular disease (number, %)	2 (2.4)
known arterial aneurysm (number, %)	1 (1.2)
history of thromboembolic disease (number, %)	2 (2.4)
beta-blocker (number, %)	72 (86.7)
alfa-blocker (number, %)	28 (33.7)
diuretic (number, %)	37 (44.6)
Ca-blocker (number, %)	41 (49.4)
ACE-inhibitor (number, %)	28 (33.7)
antiplatelet (number, %)	38 (45.8)
insulin (number, %)	15 (18.1)
oral hypoglycemic (number, %)	6 (7.2)
statin (number, %)	51 (61.4)
steroid (%)	73 (87.9)
cyclosporin A (%)	11 (13.3)
tacrolimus (%)	62 (74.7)
mycophenolate mofetil (%)	71 (85.5)
azathioprine (%)	3 (3.6)
sirolimus/everolimus (%)	3 (3.6)

**Table 2:** Dialysis fistula aneurysm characteristics.

Type of Dialysis Fistula		
	radio-cephalic (*n*, %)	36 (43.4)
	brachio-cephalic (*n*, %)	31 (37.3)
	Gracz (*n*, %)	7 (8.4)
	brachio-basilic (*n*, %)	3 (3.6)
	composite vein + PTFE (*n*, %)	6 (7.2)
**Aneurysm Characteristics**		
	location (forearm/arm)	43/40
	diameter (mean, range) (mm)	27.4 (11.1–55)
	length (mean, range) (mm)	144.6 (10–350)
**Indication for Surgery**		
	fistula thrombosis (%)	4.8
	hyperkinetic flow (%)	57.8
	rupture risk (%)	10.8
	no need for dialysis fistula (%)	56.6
	ineffective dialysis (%)	9.6
**Balazs-Bjork Scale**		
	type I (%)	63.8
	type II (%)	25.3
	type III (%)	7.2
	type IV (%)	3.6
**Valenti Scale**		
	type 1 (%)	63.8
	type 2 (%)	6
	type 3 (%)	27.7
	type 4 (%)	2.4

**Table 3:** Complications after dialysis fistula reconstruction in kidney transplant recipients (KTx group) and dialyzed patients (ESRD group).

	KTx Group (83 Patients)	ESRD (123 Patients)
outflow stenosis	6 (7.2%)	41 (33.3%)
Hematoma	3 (3.6%)	4 (3.3%)
Thrombosis	2 (2.4%)	41 (33.3%)
new aneurysm formation	2 (2.4%)	15 (12.2%)
Bleeding	2 (2.4%)	0 (0.0%)
Infection	1 (1.2%)	10 (8.1%)
Lymphocele	1 (1.2%)	9 (7.3%)
inflow stenosis	0	6 (4.9%)
distal ischemia	0	1 (0.8%)
total complications	11 patients (13.3%)	62 patients (50.4%)

**Table 4:** Morphology and biochemical parameters—dialysis fistula aneurysm reconstruction in the kidney transplant recipients and the end-stage renal disease patients.

	KTx Patients	ESRD Patients	*p*-Value
WBC (×10^9^/L)	6.5	5.6	**0.015**
RBC (×10^12^/L)	4.2	3.7	**0**
Hb (g/dL)	12.3	11.2	**0**
Hct (%)	38.45	35.2	**0**
MCV (fl)	90.45	95.2	**0**
MCHC (g/dL)	32.1	32	0.26
RDW-CV (%)	14.4	14.8	0.135
PLT (×10^9^/L)	190.5	190	0.477
PDW (fl)	11.35	12.1	0.297
MPV (fl)	10.2	10.35	0.45
Neutrophils (×10^9^/L)	4.63	3.57	**0.002**
Neutrophils (%)	67.3	61.8	**0.015**
Lymphocytes (×10^9^/L)	1.13	1.22	0.069
Lymphocytes (%)	21.4	24.2	0.105
Monocytes (×10^9^/L)	0.62	0.53	**0.008**
Monocytes (%)	8	10.5	**0**
Eosinophiles (×10^9^/L)	0.075	0.18	**0**
Eosinophiles (%)	1.25	2.8	**0**
Basophiles (×10^9^/L)	0.01	0.03	**0.04**
Basophiles (%)	0.4	0.6	**0**
Urea (mg/dL)	56	77	**0**
Creatinine (mg/dL)	1.58	5.61	**0**
eGFR (mL/min)	42	9	**0**
INR	1.07	1.09	0.148
APTT (s)	29.2	30.1	0.075
K+ (mmol/L)	4.59	4.83	**0.001**
Na+ (mmol/L)	140	139	**0.014**
Glucose (mg/dL)	100	98	0.424

Abbreviations: WBC—white blood cell count; RBC—red blood cell count; Hb—Hemoglobine; Hct—Hematocrit; MCV—Mean corpuscular volume; MCHC—Mean corpuscular hemoglobin concentration; RDW-CV—Red cell distribution width; PLT—platelet count; PDW—platelet distribution width; MPV—Mean platelet volume; eGFR—estimated glomerular filtration rate; INR—international normalized ratio; APTT—activated partial thromboplastin time; K+—potassium; Na+—sodium. Bold values represent *p* values < 0.05.

**Table 5:** Complications and patency rates in kidney recipient (KTx) and dialyzed control groups (ESRD) after dialysis fistula aneurysm excision, excision combined with primary reconstruction, excision combined with PTFE bypass.

Group	Complications	12-Month Primary Patency	12-Month Primary Assisted Patency	12-Month Secondary Patency	36-Month Primary Patency	36-Month Primary Assisted Patency	36-Month Secondary Patency
KTX-excision	1 (2%)	NA					
ESRD-excision	1 (9.09%)	NA					
KTx-excision+primary reconstruction	2 (20%)	100%	100%	100%	83.3%	83.3%	83.3%
ESRD-excision+primary reconstruction	5 (50%)	70%	100%	100%	40%	57.1%	57.1%
KTx-excision+PTFE bypass	8 (34.8%)	85%	90%	95%	64.3%	76.9%	83.3%
ESRD-excision+PTFE bypass	50 (53.2%)	79.2%	86.6%	91.7%	26..6%	37.9	50%

Data presented as the number of patients (percentage of the group). (NA—non-applicable).

**Table 6:** Morphology and biochemical parameters in the kidney transplant patients—all-native vessel vs. PTFE bypass creation.

	Aneurysm Excision + PTFE	Primary Reconstruction	*p*-Value
WBC (×10^9^/L)	6.3	6.06	0.453
RBC (×10^12^/L)	4.02	3.42	0.101
Hb (g/dL)	11.8	10.05	0.168
Hct (%)	37.4	31.2	**0.048**
MCV (fl)	92.3	92.3	0.477
MCHC (g/dL)	31.5	32.3	0.178
RDW-CV (%)	14.8	14.45	0.105
PLT (×10^9^/L)	180	192.5	0.362
PDW (fl)	11.5	11	0.116
MPV (fl)	10.4	9.85	**0.027**
Neutrophils (×10^9^/L)	4.59	3.85	0.403
Neutrophils (%)	68.6	60.3	0.101
Lymphocytes (×10^9^/L)	1.08	1.55	**0.015**
Lymphocytes (%)	17.7	21.9	0.115
Monocytes (×10^9^/L)	0.47	0.51	0.182
Monocytes (%)	7.3	8.7	0.182
Eosinophiles (×10^9^/L)	0.04	0.17	0.005
Eosinophiles (%)	0.5	2.1	0.009
Basophiles (×10^9^/L)	0.01	0.04	0.057
Basophiles (%)	0.3	0.6	0.009
Urea (mg/dL)	82	96.5	0.105
Creatinine (mg/dL)	2.2	5.4	0.004
eGFR (mL/min)	29	11.3	0.003
INR	1.05	1.11	0.056
APTT (s)	29	32.2	0.019
K+ (mmol/L)	4.6	5.2	0.002
Na+ (mmol/L)	140	139.5	0.461
Glucose (mg/dL)	100	100.5	0.355

Abbreviations: WBC—white blood cell count; RBC—red blood cell count; Hb—Hemoglobine; Hct—Hematocrit; MCV—Mean corpuscular volume; MCHC—Mean corpuscular hemoglobin concentration; RDW-CV—Red cell distribution width; PLT—platelet count; PDW—platelet distribution width; MPV—Mean platelet volume; eGFR—estimated glomerular filtration rate; INR—international normalized ratio; APTT—activated partial thromboplastin time; K+—potassium; Na+—sodium. Bold values represent *p* values < 0.05.

## Data Availability

The data presented in this study are available on request from the corresponding author.
